# Effectiveness of Physio Acoustic Sound (PAS) therapy in demented nursing home residents with nocturnal restlessness: study protocol for a randomized controlled trial

**DOI:** 10.1186/1745-6215-13-34

**Published:** 2012-04-11

**Authors:** Arnoldien J van Os, Leelie Aziz, Dorus Schalkwijk, Jos MGA Schols, Rob A de Bie

**Affiliations:** 1 Zorgspectrum Het Zand, Hollewandsweg 17, Zwolle 8014BE, The Netherlands; 2 Maastricht University, PO Box 616, Maastricht 6200MD, The Netherlands; 3 Department of General Practice and Department of Health Services Research, Caphri-School for Public Health and Primary Care, Maastricht, The Netherlands; 4 Department of Epidemiology and Institute for Education, Caphri-School for Public Health and Primary Care, Maastricht, The Netherlands

**Keywords:** Dementia, Sleep, Actiwatch, Physio acoustic sound, Nursing home, Nocturnal restlessness

## Abstract

**Background:**

Many older people with neuropsychiatric disorders such as Alzheimer's disease and frontotemporal dementia suffer from sleeping problems and often show nocturnal restlessness. Professionals and informal carers face considerable problems in solving these problems. Attempts to diminish these problems with medication in a safe and responsible manner have proven hardly effective or not effective at all. Therefore, nowadays the focus lies more on non-pharmacological solutions, for example by influencing environmental factors. There are indications that treatment with low-frequency acoustic vibrations, that is Physio Acoustic Sound (PAS) therapy, has a positive effect on sleeping problems. Therefore we study the effectiveness of PAS therapy in demented patients with nocturnal restlessness.

**Methods:**

In a randomized clinical trial, 66 nursing home patients will be divided into two groups: an intervention group and a control group. For both groups nocturnal restlessness will be measured with actiwatches during a period of six weeks. In addition, a sleep diary will be filled in.

For the intervention group the baseline will be assessed, in the first two weeks, reflecting the existing situation regarding nocturnal restlessness. In the next two weeks, this group will sleep on a bed identical to their own, but with a mattress containing an in-built PAS device. As soon as the patient is lying in bed, the computer programme inducing the vibrations will be switched on for the duration of 30 min. In the last two weeks, the wash-out period, the measurements of the intervention group are continued, without the PAS intervention.

During the total study period, other relevant data of all the implied patients will be recorded systematically and continuously, for example patient characteristics (data from patient files), the type and seriousness of the dementia, occurrence of neuropsychiatric symptoms during the research period, and the occurrence of intermittent co-morbidity.

**Discussion:**

If PAS therapy turns out to be effective, it can be of added value to the treatment of nocturnal restlessness in demented patients. Non-pharmacological PAS therapy is not only safe and patient-friendly, but it can also be widely used in a simple and relatively inexpensive way, both in institutions such as nursing homes and residential homes for the elderly, and at home. Ultimately, this may lead to a decrease in the frequent and still common use of psychotropic drugs. In addition, care needs of demented patients also may decrease as well as the number of preventable admissions to care institutions.

**Trial registration:**

Netherlands Trial Register (NTR): NTR3242

## Background

Many older people with neuropsychiatric disorders such as Alzheimer's disease [1] and frontotemporal dementia [2] often suffer from sleeping problems, especially nocturnal restlessness. The concept of nocturnal restlessness can be described as a disturbance of the normal sleeping pattern, manifesting itself in a certain way of motor and/or verbal behavior. In general, nocturnal restlessness should be placed in a wider context of behavioral disorders, that are often associated with dementia [3]. Common types of nocturnal restlessness include a change or even a reversal of the day/night rhythm, high motor activity levels during sleep, a frequent alteration of sleeping phases and a frequent crying out and/or wandering during the night. This disturbance of normal sleep may lead to or coincide with serious inconvenience and stress, not only for the patients themselves but also for their informal carers, which is an especially important reason to transfer (or admit early) a patient to a nursing home [4].

### Treatment of sleep disturbances in dementia

Nocturnal restlessness as well as other behavioral problems associated with dementia, are still mostly being treated with psychotropic medication such as hypnotics and antipsychotics in nursing homes as well as at home. The results of such treatments are often disappointing and negative side effects occur frequently. Research conducted in nursing homes has even demonstrated that this pharmacological approach has more adverse than positive effects [5,6].

Adequate control of the day/night rhythm by influencing lifestyle and environmental factors may sometimes lead to better results [4-10]. This finding stimulates further research on other, preferably non-pharmacological treatment options for the problems of nocturnal restlessness in dementia.

### Effectiveness of Physio Acoustic Sound therapy

A new non-pharmacological treatment option was found by chance by the staff of Zandhove, the central nursing home of Zorgspectrum Het Zand in Zwolle, the Netherlands. It arose from the successful therapeutic application of acoustic vibrations in order to reduce pain. This Physio Acoustic Sound (PAS) therapy was originally developed in Finland, where it has been used successfully for several decades, especially in patients with pain, stress, and rigidity.

The therapy is based on practical experience as well as research, both illustrating that low-frequency acoustic vibrations (27-113 Hz) can have positive effects on several symptoms. For instance, in an intervention period of five weeks, PAS therapy has led to a decrease in self-wounding, stereotype, and aggressive self-destructive behavior in patients with autism and development disorders [11]. Furthermore, a PAS therapy programme for the elderly has proven to improve their general wellbeing and functional capacity: bone formation and mobility improved, cholesterol levels were lower, and blood circulation was stimulated [12]. In another study, PAS therapy caused patients to be more relaxed with reduction of their pain symptoms [13,14]. The therapy also has shown positive short-term effects on motor symptoms in patients with Parkinson's disease, for example a decrease in rigidity, bradykinesia, tremor, and an increase in step length [15].

Of the many observed effects of PAS therapy, the following have now been officially recognized by the American Food and Drug Administration (Device Classification Name: vibrator, therapeutic; 510(k) nr. K934886, reg. nr. 890.5975; d.d. 06-09-1994): relaxation, reducing pain, and stimulating blood circulation.

Most of the results of PAS therapy have been obtained by treatment in a so-called PAS therapy chair. The chair contains acoustic equipment that generates a limited range of low-frequency vibrations (27-113 Hz). Controlled by computer programmes, these vibrations are led through the chair in a wave sequence. The chairs are now available in several countries, including the Netherlands, and are used for several treatment purposes [16].

### PAS therapy in sleep disturbances in dementia

**Figure 1:**
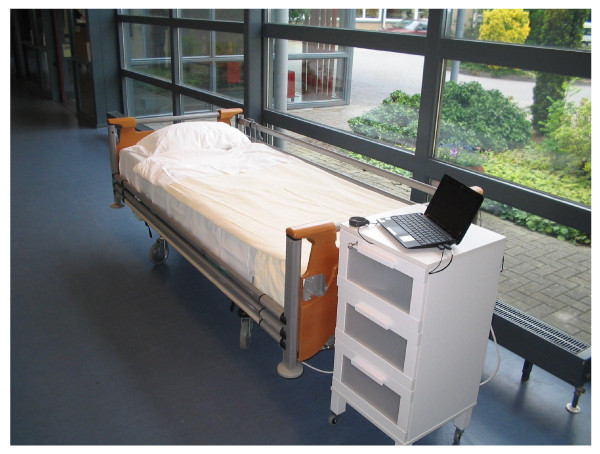
**High-dependency bed with PAS mattress and night table with computer which is normally locked up in the night table**.

Many of the effects of PAS therapy mentioned above have also been observed in daily practice in the nursing home Zandhove of Zorgspectrum Het Zand. Moreover, patients indicated that they were sleeping better after treatment in the PAS therapy chair. To investigate whether this particular effect also applies to demented elderly people, for whom nocturnal restlessness is a common cause of sleeping disorders, the same technique as in the chair was recently built-in in mattresses. These mattresses can be used on a standard high-dependency bed as used in most long-term care settings (Figure 1).

## Methods and design

### General objective

The aim of this study is to test, by means of a randomized controlled trial, the hypothesis that application of PAS therapy with low-frequency acoustic vibrations to demented patients in nursing homes will lead to a significant decrease of nocturnal restlessness.

### Participants

The research population consists of demented nursing home patients residing in one of the five psychogeriatric nursing wards of Zorgspectrum Het Zand. Each ward has about 30 patients, who each have a single bedroom and share a common living room (8 to 10 people).

### Inclusion and exclusion criteria

Patients are included when diagnosed with the DSM-IV definition of dementia and who have been residing in the nursing home for a minimum of two months. Exclusion criteria are (1) the presence of (high risk of) a pressure sore, that is an indication for the preventive use of a different mattress, for example an alternative mattress to prevent pressure ulcers; (2) acute illness, for example pneumonia, exacerbation of heart failure, a recent stroke, and so on; and (3) a pacemaker, which could possibly interfere with the PAS instruments.

### Interventions

Patients will be randomized over an intervention group and a control group. The patients in the control group will sleep on their own high-dependency bed during the three periods of two weeks each. The patients in the intervention group will sleep, after the initial baseline period of two weeks, on a high dependency bed identical to their own for the second period of two weeks. The mattress of this bed, however, contains the same technique as the PAS therapy chair and will be controlled by an external computer. As the intervention can be controlled perfectly and safely via the computer regarding frequency, duration and intensity, the technique is considered suitable for this research. In the third and last (wash-out) period of two weeks they sleep again in their own bed.

The computer programme controlling the acoustic vibrations of the mattress will be switched on daily by the nursing staff during a period of two weeks. This will be done as soon as the patient is lying in bed. The programme will switch off automatically after 30 min. So as to not contaminate the results by the presence of the staff, the staff member will leave the patient's bedroom as soon as the mattress is switched on. The frequencies used in the programme lie between 27 and 40 Hz and will vary every two or three minutes.

These frequency settings of the intervention have been chosen after advice from Finnish professionals who have many years of experience with this intervention. The design of the programme used in this intervention was originally aimed at reducing pain, and the varying frequencies were based on Melzak and Wall's gate control theory [17].

The other therapeutic applications of PAS therapy described above, whose effectiveness has already been proven, are all based on this design as well.

### Sample size

In this study, it is assumed that the nocturnal restlessness in patients in the control group will remain stable, with a frequency of 80% episodes of nocturnal restlessness [18]. By applying PAS therapy, a conservative decrease of nocturnal restlessness of 30% is expected, resulting in a restlessness frequency of 50%. Taking into account a probability value of 5% (alpha), a power of 80% (1-beta), and a drop-out rate of 10%, 66 patients are needed in total, that is 33 in the intervention group and 33 in the control group.

### Randomization

By means of blinded block randomization with group sizes of six, the randomization scheme will determine which patients will be placed in the intervention group and the control group. The intervention is not blinded because it is visible for the caregiver (box with computer below the bed and faintly audible (a very low buzz).

### Procedure

After including the patients for participation in the study, they will be divided into an intervention and control group by means of randomization. We have not opted for a crossover design, as it cannot be estimated how long the wash-out period will be [19]. For the sake of the validity of the results to be obtained, a period of six weeks (2 + 2 + 2) has been chosen for the intervention group as well as for the control group. During this total period, both groups of demented patients will continuously wear an actiwatch (respironics type AW2) on the non-dominant wrist. Practice has shown that this watch hardly causes any nuisance or no nuisance at all.

In addition, a daily sleep diary will be kept by the nursing staff for all participants.

In the first two weeks, the baseline situation (T0) of the intervention group and control group will be measured by means of the actiwatch. In the next two weeks, as the intervention is executed in the intervention group, in both groups the actiwatch measurements will be continued (T1). In the last two weeks the wash-out of the intervention will be measured and again in both groups the actiwatch measurements will be continued (T2).

During the whole period, the doors of the bedrooms of all participants in both groups will remain closed during the night, in order to prevent disruptions. Yet, if a patient or staff member enters/leaves the room, this will be noted in the sleep diary.

After the study period of six weeks, the actiwatches will be read out. The collected data per patient will be recorded, together with the other relevant data. These include registered changes in the situation of the patients during the study period, for example (1) possible additional co-morbid illness(es) that may lead to a change in the level of motor restlessness or in the normal day/night pattern of the patient (for example pneumonia, delirium, urinary retention, constipation), or (2) changes in the use of psychotropic drugs, therapies, and/or use of restraints.

A schematic representation of the procedure can be found in Table 1.

**Table 1:** Measurements overview

Moment	T0	Baseline	T1	Baseline	T2	Intervention	T3	Intervention	T4	Wash-out	T5	Wash-out	T6
Week	0	1		2		3		4		5		6	
Actiwatch		Continuous		Continuous	*Charging*	Continuous		Continuous	*Charging*	Continuous		Continuous	
NPI-Q					T2				T4				T6
BANS-S	T0												
Patient characteristics	T0												
Sleep diary		Continuous		Continuous		Continuous		Continuous		Continuous		Continuous	
Status examination			T1		T2		T3		T4		T5		T6

T0, the week previous to the measuring period: complete BANS-S and make an inventory of patient characteristics (CIRS); T1, status examination; T2, end of baseline period: complete NPI-Q and status examination; T3, status examination; T4, end of intervention period: complete NPI-Q and status examination; T5, status examination; T6, end of wash-out period: complete NPI-Q and status examination. Actigraphy is taking place continuously. At T2 and T4, the actiwatch will be charged for a short period (ca. 2 h).

A qualitative status examination will take place weekly (changes in medication, therapy, medical condition, means and measures, restlessness). The sleep diary will be filled in daily during the whole measuring period.

### Data collection

First, the following patient characteristics will be collected for both groups: age, gender, type of dementia, co-morbidity (by means of CIRS [20]), incontinence Yes or No, the use of restraints (for example fixation belts), medication (including psychopharmacological drugs), and other therapies.

In addition, the Bedford Alzheimer Nursing Severity Scale (BANS-S) [21] will be applied by the psychologist together with the nursing staff to get an impression of the severity of dementia. This validated scale contains seven items to assess functional and cognitive capacities as well as pathological symptoms. These items, scaled 1 to 4, are summed. The overall score ranges from 7 (no impairment) to 28 (complete impairment).

Although the traditional 'gold standard' assessing the quality of the sleep is polysomnography (PSG), it is difficult to apply PSG to patients with a moderate to serious form of dementia [22,23]. Therefore, in studies like this, often more feasible assessment methods are used, like observation-oriented techniques and actigraphy. Actigraphy is a less invasive method than PSG, offering a reliable way to estimate sleep/wake parameters including nocturnal restlessness [24]. In this study we will use both actigraphy and sleep diaries.

The nocturnal restlessness will be measured by validated actigraphy [25-27] in the intervention group as well as in the control group on the basis of the following sleep/wake parameters [28,29]: (1) nocturnal restlessness: average motor activity during the night; (2) sleep efficiency: the number of hours the patient is lying in bed and actually sleeping; (3) wake bouts: the number of episodes the patient is awake during the night; (4) inter-daily stability: the degree of stability in activity patterns over longer periods of time; (5) intra-daily variability: the degree of inconstancy in rest and activity during a day [28,29].

When considering nocturnal restlessness it is important to take measurements during a period of at least two weeks. In this way, the statistic power will increase [30].

To determine the degree to which the actimeters respond to the acoustic vibrations of the mattress themselves, a control actimeter is placed on the mattress in advance.

In addition, other relevant sleep observation data of every patient will be written down in a sleep diary, in which nocturnal restlessness and the day/night rhythm, as observed by the nursing staff will be recorded.

As shown in previous research, this 'subjective' information will optimize the 'objective' information gained by using the actimeters [31]. The nursing staff will receive instructions on how to use the actiwatch, how to fill in the sleep diary, and how to operate the computer.

Finally, it is important to carefully monitor the changes in behavior of the participating demented residents, over the total study period. Therefore, a validated Neuropsychiatric Inventory assessment (NPI-NH) [32] will be performed in weeks 2, 4, and 6 by a psychologist together with a nursing staff member who knows the patient well. The NPI-NH contains 10 behavioral aspects and two types of neuro-vegetative changes, that is delusions, hallucinations, agitation, depression/dysphoria, anxiety, euphoria/elation, apathy/indifference, disinhibition, irritability/lability, aberrant motor behavior, night-time disturbances, and appetite/eating change. This inventory will provide information on possible coexisting neuropsychiatric symptoms in patients with Alzheimer disease or other dementia syndromes.

Both the frequency and severity of each symptom are rated on a four- (1-4) and three-point (1-3) Likert scale, respectively. A separate score can be calculated for each symptom by multiplying the frequency and severity scores, results ranging from 0 to 12 for each symptom. A total score can be obtained by summing the 12 frequency and severity scores, yielding total scores that range from 0 to 144 [33].

### Adverse effects

The study will be terminated if (1) the patient resists by verbally and/or non-verbally putting up resistance when using the intervention mattress and/or the actiwatch to a higher degree than usual in situations deviating from the daily routine; and (2) one of the previously mentioned exclusion criteria is met during the intervention period.

As demented residents often cannot express their own will themselves, the legal representative (mostly a family member), together with the nursing staff, nursing home physician, and/or psychologist, will assess whether resistance to the intervention applies to the patient.

### Approval

This study follows the Dutch Medical Research Involving Human Subjects Act's (WMO) and the Helsinki Declaration's principles, meaning that the (legal) representatives of all demented patients will sign a written informed consent, stating also that participation can be withdrawn at any time, whenever the patient shows resistance, without any negative consequences concerning their current or future medical treatment. The Medical Ethical Committee of Maastricht University has granted ethical approval (protocol 2011 nr. NL35287.068.11).

### Statistical analysis

In order to process the data to test our hypothesis, the statistical software programme SPSS-19 [34] will be used. First, data will be checked for missing values and normal distribution. It will be examined whether there is a significant difference in both nocturnal restlessness and degree of neuropsychiatric symptoms between the intervention group and the control group.

To analyze the actigraphy variables and the variables measured by means of NPI-NH (T0, T1, and T2) integrally, a multilevel regression model (Mixed Models) will be used. In this study, the Basic Two-Level Regression Model [35] will be used, in which the actigraphy variables and NPI-NH variables are the lowest levels (that is dependent variable) and the research participants the highest level (independent variable).

The multilevel analysis enables us to calculate the degree of nocturnal restlessness and neuropsychiatric symptoms despite the unequal number of observations per individual (missing values/dropouts). This means that all data will be included in the analysis, including the data of participants who will meet stop criteria during the investigation and for whom the study has to be terminated early.

If there is interaction effect between time and group, a post-hoc analysis will be performed, taking into account that T0-T1 indicates PAS therapy effects and that T1-T2 indicates the long-term effects of PAS therapy. The analyses will be corrected for basic differences in the characteristics of the patients belonging to the intervention group and the control group respectively. The data in the sleep diaries will first be coded and later analyzed separately by means of describing statistics.

### Primary outcome measures

The objectively and subjectively measured difference in nocturnal restlessness between T0-T1-T2. Objectively, the nocturnal restlessness measured during six weeks by means of an actiwatch validated for sleep research with the sleep/wake parameters mentioned above.

Subjectively, nocturnal restlessness determined on the basis of a sleep diary developed for this study, and filled in by the nursing staff.

### Secondary outcome measures

The secondary outcome measure in this study involves an analysis of the prevalence and the degree of change in overall neuropsychiatric symptoms of the nursing home patients taking part in the study.

## Discussion

In general, professionals and informal caregivers often face considerable difficulties in solving behavioral problems present in demented people. In this study we focus on the problem of nocturnal restlessness, often leading to a diminished quality of life of the patients involved, and sometimes a severe burden for the patient's environment. Attempts to solve this problem adequately and safe with medication have proven to be hardly effective or not effective at all [5].

That is the most important and underlying reason for research on the effectiveness of non-pharmacological interventions. This study about the effectiveness of the PAS therapy, a non-pharmacological treatment option for nocturnal restlessness in demented people, is an example of this type of studies. PAS therapy is safe and patient-friendly and has not led to undesired side effects or reports of adverse effects in other applications.

The design chosen for this study has some limitations. For instance, it is not possible to carry out a double-blinded study, as the PAS mattress involves a light buzzing and is controlled by a computer that is built in the night table next to the bed.

However, given the setting and the nature of the group of residents, the risk of contamination can be considered to be small.

Furthermore, actigraphy is particularly suitable for measuring nocturnal restlessness, but less suitable for measuring periods during which the patient lies awake quietly [27].

In addition, it must be noted that when it comes to writing down data in the sleep diaries, the researchers depend on the nursing staff's compliance. Therefore, the nursing staff has received explicit instructions, and the researchers regularly check whether these instructions are being followed. Nevertheless, in daily practice of nursing home care, it is impossible to completely take into account all relevant factors.

Despite these limitations, if proven effective, this form of PAS therapy can be widely introduced in a simple and relatively inexpensive manner in order to reduce nocturnal restlessness in demented people. The therapy can be applied in institutions, for example nursing homes and residential homes for the elderly, as well as at home.

Ultimately, this may lead to a decrease in the frequent and still common use of often harmful psychotropic drugs.

In a more general sense, the care needs of demented people may decrease as well as the number of preventable admissions to care institutions [36].

## Trial status

The study started recruiting participants in July 2011. This is still ongoing. Soon after the first recruitments, the interventions and the measurements started as well. We expect the results to be published at the end of 2013.

## Abbreviations

BANS-S: Bedford Alzheimer nursing severity scale; BOPZ: Wet bijzondere opnemingen in psychiatrische ziekenhuizen (Psychiatric Hospitals Compulsory Admissions Act); CIRS: Cumulative illness rating scale; NPI-NH: Neuropsychiatric Inventory - Nursing home version; PAS: Therapy Physio Acoustic Sound therapy; PSG: Polysomnography; WMO: Wet medisch-wetenschappelijk onderzoek met mensen (Medical Research Involving Human Subjects Act).

## Competing interests

The authors declare that they have no competing interests.

## Authors' contributions

AJO is initiator of the study, obtained funding, participated in the study design and trial coordination, and drafted the manuscript. LA and DS participated in the study design, patient recruitment, and trial coordination. JMGAS and RAB participated in the study design, and supervised and participated in writing the manuscript. All authors have read and approved the final manuscript.
